# Molecular Modeling, Synthesis and Biological Evaluation of *N*-Phenyl-4-Hydroxy-6-Methyl-2-Quinolone-3-CarboxAmides as Anticancer Agents

**DOI:** 10.3390/molecules25225348

**Published:** 2020-11-16

**Authors:** Dima A. Sabbah, Shaima’ E. Hasan, Reema Abu Khalaf, Sanaa K. Bardaweel, Rima Hajjo, Khalid M. Alqaisi, Kamal A. Sweidan, Aya M. Al-Zuheiri

**Affiliations:** 1 Department of Pharmacy, Faculty of Pharmacy, Al-Zaytoonah University of Jordan, P.O. Box 130, Amman 11733, Jordan; shaimaa94@hotmail.com (S.E.H.); reema.abukhalaf@zuj.edu.jo (R.A.K.); r.hajjo@zuj.edu.jo (R.H.); ayaalzuhyri@gmail.com (A.M.A.-Z.); 2 Department of Pharmaceutical Sciences, School of Pharmacy, University of Jordan, Amman 11942, Jordan; s.bardaweel@ju.edu.jo; 3 Department of Medical Laboratory Sciences, Faculty of Allied Medical Sciences, Al-Ahliyya Amman University, Amman 19328, Jordan; k.alqaisi@ammanu.edu.jo; 4 Pharmacological and Diagnostic Research Centre (PDRC), Faculty of Pharmacy, Al-Ahliyya Amman University, Amman 19328, Jordan; 5 Department of Chemistry, The University of Jordan, Amman 11942, Jordan; k.sweidan@ju.edu.jo

**Keywords:** anticancer, colon cancer, PI3Kα, AKT, docking, quinolone-3-carboxamide

## Abstract

The emergence of phosphatidylinositol 3-kinase (PI3Kα) in cancer development has accentuated its significance as a potential target for anticancer drug design. Twenty one derivatives of *N*-phenyl-4-hydroxy-6-methyl-2-quinolone-3-carboxamide were synthesized and characterized using NMR (^1^H and ^13^C) and HRMS. The derivatives displayed inhibitory activity against human epithelial colorectal adenocarcinoma (Caco-2) and human colon cancer (HCT-116) cell lines: compounds **8** (IC_50_ Caco-2 = 98 µM, IC_50_ HCT-116 = 337 µM) and **16** (IC_50_ Caco-2 = 13 µM, IC_50_ HCT-116 = 240.2 µM). Results showed that compound **16** significantly affected the gene encoding AKT, BAD, and PI3K. The induced-fit docking (IFD) studies against PI3Kα demonstrated that the scaffold accommodates the kinase domains and forms H-bonds with significant binding residues.

## 1. Introduction

Phosphatidylinositol 3-kinases (PI3Ks) phosphorylate the hydroxyl moiety on position 3 (3-OH) of inositol rings, producing phosphatidylinositol 3,4,5 triphosphates (PIP_3_). The generated triphosphates induce downstream messengers and signaling receptors such as protein kinase B (AKT), and consequently promote cell proliferation and division [1,2].

The phosphatase and tensin homolog (PTEN) dephosphorylate the 3-OH group of PIP_3_ and adversely control PI3Ks [2,3]. PI3Ks are classified into three classes (I, II, and III) based on their substrate binding and primary structures. Class IA PI3Ks compromises PI3Kα, β, and δ isozymes expressed by their discrete genes PIK3CA (PI3Kα), PIK3CB (PI3Kβ), and PIK3CD (PI3Kδ) [4]. Abnormal activation of the PI3Kα/AKT signaling cascade has been detected in diverse human cancers [5]. The gene PIK3CA is exaggerated, over encrypted, and mutated in many human cancers [5]. Mutations in the helical (E545K and E542K) and kinase (H1047R) domains of PI3Kα are identified in brain, breast, endometrial, and GIT cancers [6,7,8,9]. These oncogenic alterations distort PI3Kα conformation and have therefore lead to the design of selective PI3Kα inhibitors [8]. The prevalence of PI3Kα and PTEN mutations in various human cancers identify PI3Kα inhibition as a promising goal for anticancer drug design [10,11].

**Figure 1:**
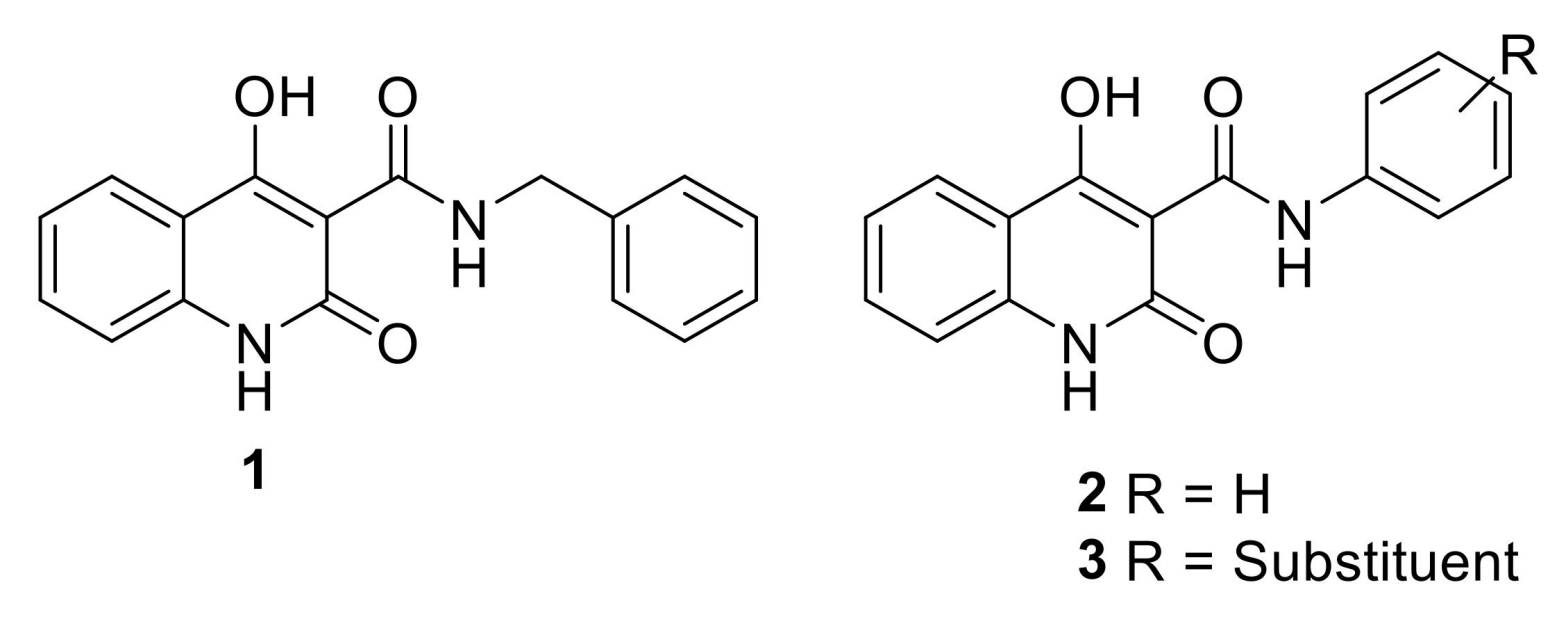
Chemical structures of **1**, **2** and **3**.

**Figure 2:**
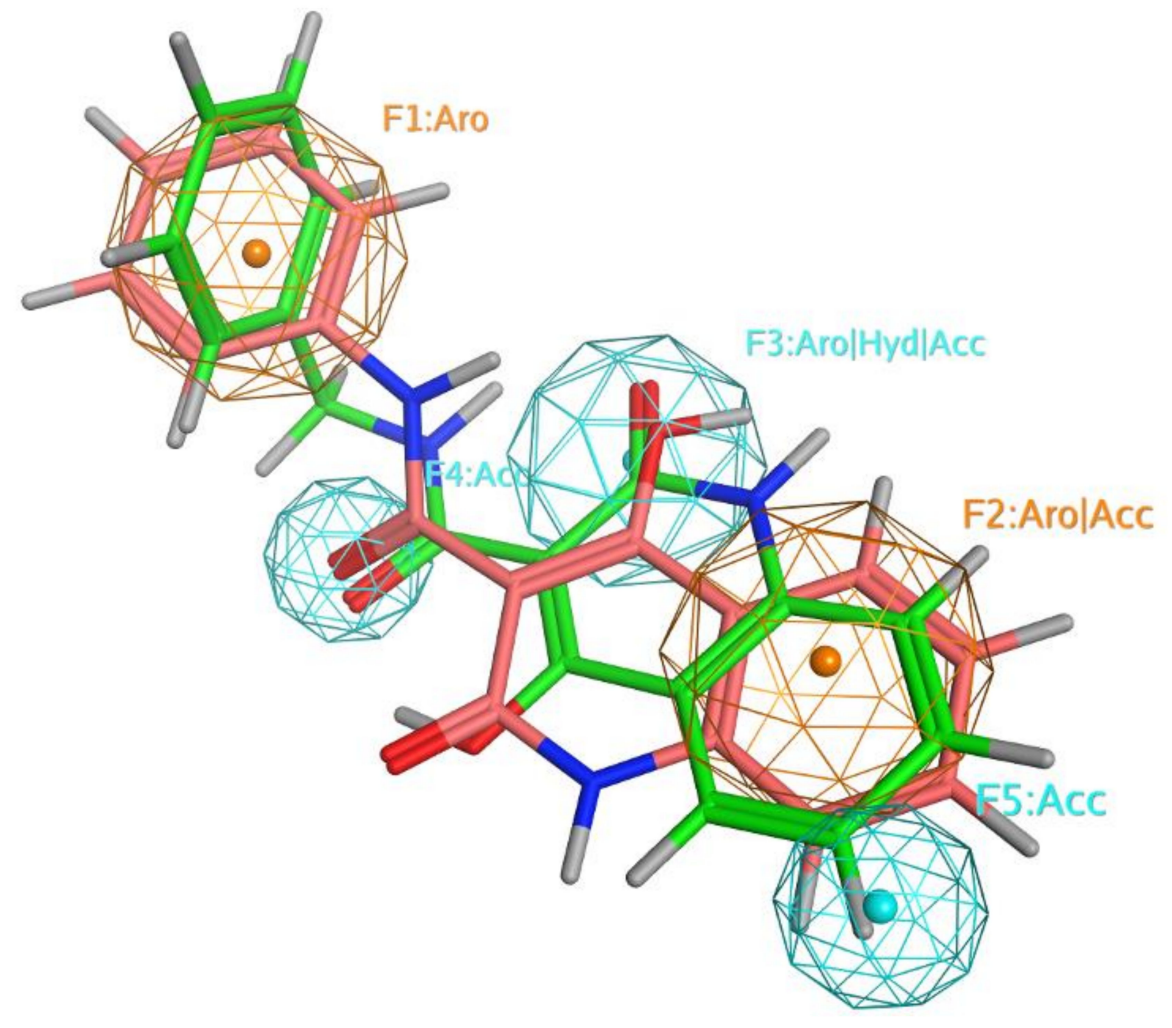
PI3Kα inhibitor pharmacophore model with (**1**) and (**2**) depicted in green and orange color, respectively. Picture made by MOE [15].

Employing pharmacophore modeling and database searching against the national cancer institute (NCI) database [12], we previously disclosed *N*-benzyl-4-hydroxy-2-quinolone-3-carboxamide (**1**) as a lead PI3Kα inhibitor against the wild-type (WT) (IC_50_ = 1.1 μM) and mutant (MUT) H1047R PI3Kα (IC_50_ = 0.73 μM) (Figure 1; Figure 2) [13]. Furthermore, we determined the structural changes between the WT and MUT PI3Kα that could be targeted to identify selective inhibitors [14].

Furthermore, we identified the key binding residues of MUT H1047R PI3Kα inhibitors through binding free energy calculations recruiting the molecular mechanics/generalized born surface area (MM/GBSA) protocol for PI3Kα molecular dynamic (MD) extracted assemblies [16]. Additionally, excessive lead optimization strategies released *N*-phenyl-4-hydroxy-2-quinolone-3-carboxamide derivatives as selective MUT H1047R PI3Kα inhibitors exemplified by **2** [17]. Lately, we released a series of *N*-substituted 4-hydroxy-2-quinolone-3-carboxamides that suppressed human colon carcinoma (HCT116) cell line proliferation and enhanced apoptosis through inducing caspase-3 activity and destructing cellular DNA illustrated by **3** [18]. Our ceaseless research has uncovered diverse scaffolds [19,20,21,22,23,24] targeting PI3Kα.

In this context, we designed and synthesized a series of *N*-phenyl-4-hydroxy-6-methyl-2-quinolone-3-carboxamides to address the effect of incorporating a methyl moiety on the biological activity and to better understand the structural activity relation (SAR) of this series. This work delineates the design and synthesis of novel *N*-phenyl-4-hydroxy-6-methyl-2-quinolone-3-carboxamides employing structure-based drug design and molecular modeling tactics. Biological evaluation of the prepared compounds along with pan PI3K inhibitors (LY294002) has been investigated in vitro against human epithelial colorectal adenocarcinoma (Caco-2) and human colon carcinoma (HCT-116) cell lines.

## 2. Results and Discussion

### 2.1. Chemistry

**Scheme 1:**
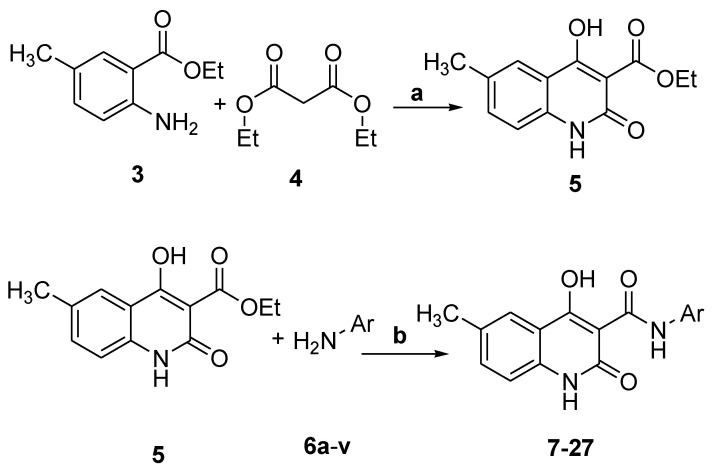
Conditions: (**a**): (i) NaOC_2_H_5_, DMSO, 130–140 °C, 72 h. (ii) 0.3M HCl (**b**): (i) THF, DMF, 150 °C, 48 h.

Target compounds (**7**–**27**) were designed to probe the influence of introducing different functionalities at the carboxamide side chain on the bioactivity of the 4-hydroxy-6-methylquinolinone scaffold. The reaction of **3** and **4** in basic medium under reflux generated the target scaffold **5** with 70% yield, as outlined in Scheme 1. Monitoring the reaction progress was applied by employing thin layer chromatography (TLC); the disappearance of the ethyl anthranilate spot indicated that the reaction was completed. Prospective compounds **7**–**27** have been synthesized by reacting **5** with an excess of the corresponding amine (ArNH_2_) using DMF under reflux, as delineated in Table 1 and Scheme 1. The absence of the limiting reactant spot (**5**) on the TLC plate indicated that the reaction was completed. Confirmation of the identity of the chemical structures was carried out using NMR and HRMS. The detailed data in the experimental section accord with the target structures.

### 2.2. Biological Evaluation of the Synthesized Compounds

In order to investigate the inhibitory activity of the verified compounds (**5**, **7**–**27**) against PI3Kα, we probed their antiproliferative activity in human epithelial colorectal adenocarcinoma (Caco-2) and human colon cancer (HCT-116) cell lines as shown in Table 2. The epithelial colorectal adenocarcinoma (Caco-2) expresses the wild-type (WT) PI3Kα [25,26,27]. The malignant (HCT-116) carcinoma encodes both wild-type (WT) and mutant (MUT) (H1047R) PI3Kα [28]. Therefore, the difference in activity against Caco-2 and HCT-116 accounts for MUT (H1047R) PI3Kα.

All examined compounds were examined against skin fibroblast cells at concentration ranges between the IC_50_ and double IC_50_ values for each compound. At each concentration point, cell viability was compared to the negative control of untreated cells. We found less than 10% growth reduction for all examined compounds at any concentration point.

The inhibitory activity against Caco-2 for compounds **14**, **15**, and **16** reveals that attaching the *p*-F moiety on the phenyl carboxamide side chain (**13**) induces activity that suggests that a hydrophobic and/or H-bond interaction mediate(s) ligand/PI3Kα complex formation. The activity of **14**, **15**, and **16** against HCT-116 indicates distinct inhibitory activity and highlights the activity of **16** in Caco-2; **16** exerted potent inhibitory activity in Caco-2. In contrast, the activity of **17**, **18**, and **19** in Caco-2 and HCT-116 shows that a steric factor might hinder their proper orientation in the binding site.

The biological data of **11**, **12**, and **13** in Caco-2 suggests that an H-bond interaction presides ligand/PI3Kα complex formation. Furthermore, comparing the activity of *p*-F (**16**) with that of *p*-OH (**11**) clarifies that a steric factor affects the accommodation of –OH in the binding domain. Additionally, contrasting the inhibitory activity of *p*-OCH_3_ (**12**) and of *p*-CH_3_ (**13**) in Caco-2 implies that an H-bond interaction might drive ligand/PI3Kα binding and/or an oxygen atom might push –CH_3_ deeply into the binding cleft. Furthermore, the inhibitory activity of **11**, **12**, and **13** in HCT-116 declares that an H-bond interaction mediates ligand/PI3Kα interaction; **11** provides an H-bond donor and acceptor, whereas **12** offers an H-bond acceptor. Interestingly, the activity of *p*-OCH_3_ (**12**) and *p*-CH_3_ (**13**) affirms that an H-bond donor, exemplified by (**11**), dominates ligand/PI3Kα complex formation and suggests that a small hydrophobic cleft encloses the –CH_3_ motif.

Comparing the activity of **7** and **8** in Caco-2 infers that a nitrogen atom in **8** might mediate the H-bond interaction with PI3Kα backbones and/or the solubility and polarity factor of pyridine might influence the compound’s distribution in the cell line. Comparing the activity of **9** and **10** in Caco-2 reveals that elongation of the carboxamide side chain by the –NH motif (**10**) improves the activity and thus infers that the H-bond might drive ligand/PI3Kα interactions. Such a result shows the low activity of **9** and underlines the significance of the –NH motif.

The biological data of **7**, **8**, **9**, and **10** in HCT-116 show that introducing the phenyl moiety on the carboxamide side chain (**7**) provokes the activity interrogating the tightness of the binding cleft, and thus, **7** accommodates the binding cleft. Contrasting the activity of **7** and **8** suggests that a hydrophobic lining encloses the phenyl motif and understates the significance of the H-bond in HCT-116. Furthermore, the activity of **9** and **10** confirms the narrowness of the binding cleft and provides a further clue to the proper orientation of **7** in the kinase domain. Furthermore, the antiproliferative activity of **7**, **8**, **9**, and **10** in Caco-2 and HCT-116 displays comparable activity for **7** and **9** and distinct activity for **8** and **10** in both cell lines.

Tailoring the derivatives with –COOH (**20**, **21**, and **22**) confirms that the H-bond mediates ligand/PI3Kα complex formation in Caco-2 on the *p*-position (**22**). Such a finding emphasizes the importance of the H-bond on the ligand/PI3Kα interaction and further explains the activity of *p*-F (**16**) and *p*-OH (**11**) in Caco-2. Additionally, the result confirms that the steric factor impedes their proper location in the binding site. Distinctly, *p*-F (**16**) and *p*-OH (**11**) exerted higher activity than that of *p*-COOH (22) in Caco-2.

The activity of **20**, **21**, and **22** in HCT-116 demonstrates that ionic and/or H-bond(s) guide(s) ligand/PI3Kα complex formation on the *m*-position (**21**). Furthermore, the activity of *m*-F (**15**), *m*-COOH (**16**), and *m*-CF3 (**18**) in HCT-116 accentuates the significance of the H-bond donor and/or ionic bond on the ligand/PI3Kα interaction. Contrasting the activity of **20** and **23** in Caco-2 and HCT-116 infers that the ester moiety improves cell membrane permeability and consequently enhances the activity.

The inhibitory activity of **24** and **25** in Caco-2 declares that tailoring the benzoic acid with –Cl (**24**) and –CH_3_ (**25**) improves the activity, suggesting that –Cl and –CH_3_ might orientate the derivatives properly in the binding site and/or provide an extra binding interaction. Contrasting the activity of **20**, **24**, and **25** in HCT-116 shows that attaching –Cl (**24**) and –CH_3_ (**25**) induces the activity, suggesting that –Cl and –CH_3_ might place the ligands suitably in the binding cleft and/or furnish an extra binding interaction.

The activity of *p*-SH (**26**) in Caco-2 provides an extra clue for the H-bond interaction on the *p*-site. Comparing the activity of *p*-OH (**11**) and p-SH (**26**) confirms that the H-bond drives the ligand/PI3Kα interaction. Moreover, contrasting the activity of *p*-SCH_3_ (**27**) to that of *p*-OCH_3_ (**12**) in Caco-2 suggests that S and O push –CH_3_ deeply into the binding site and thus in turn improves the activity. The difference in activity between **12** and **27** might be due to the H bond supplied by the O atom. It is worth noting that the series exhibited distinct antiproliferative activity in both cell lines, suggesting differences in the binding sites of WT PI3Kα and MUT (H1047R) PI3Kα, which accords with our previous data on PI3Kα [13,14,16,17,18,19,23,24].

Comparing the biological data of *p*-OH (**11**) and *p*-SH (**26**) in HCT-116 highlights the significance of the H-bond interaction on *p*-site. Furthermore, the difference in activity between *p*-OCH_3_ (**12**) and *p*-SCH_3_ (**27**) in HCT-116 shows the significance of the H bond assigned by the O atom and/or the steric interference of the S atom, which impedes the proper conformation of **27** in the binding cleft. Finally, the activity of **5** against Caco-2 and HCT-116 cell lines highlights the significance of the tailored carboxamide motif.

The explored cell lines encode both WT and MUT PI3Kαs; therefore, isolating either gene is highly recommended to confirm the inhibitory activity against purified PI3Kα. Future validations should be performed through knocking down any gene to interpret the mechanism of inhibition against each purified enzyme.

**Figure 3:**
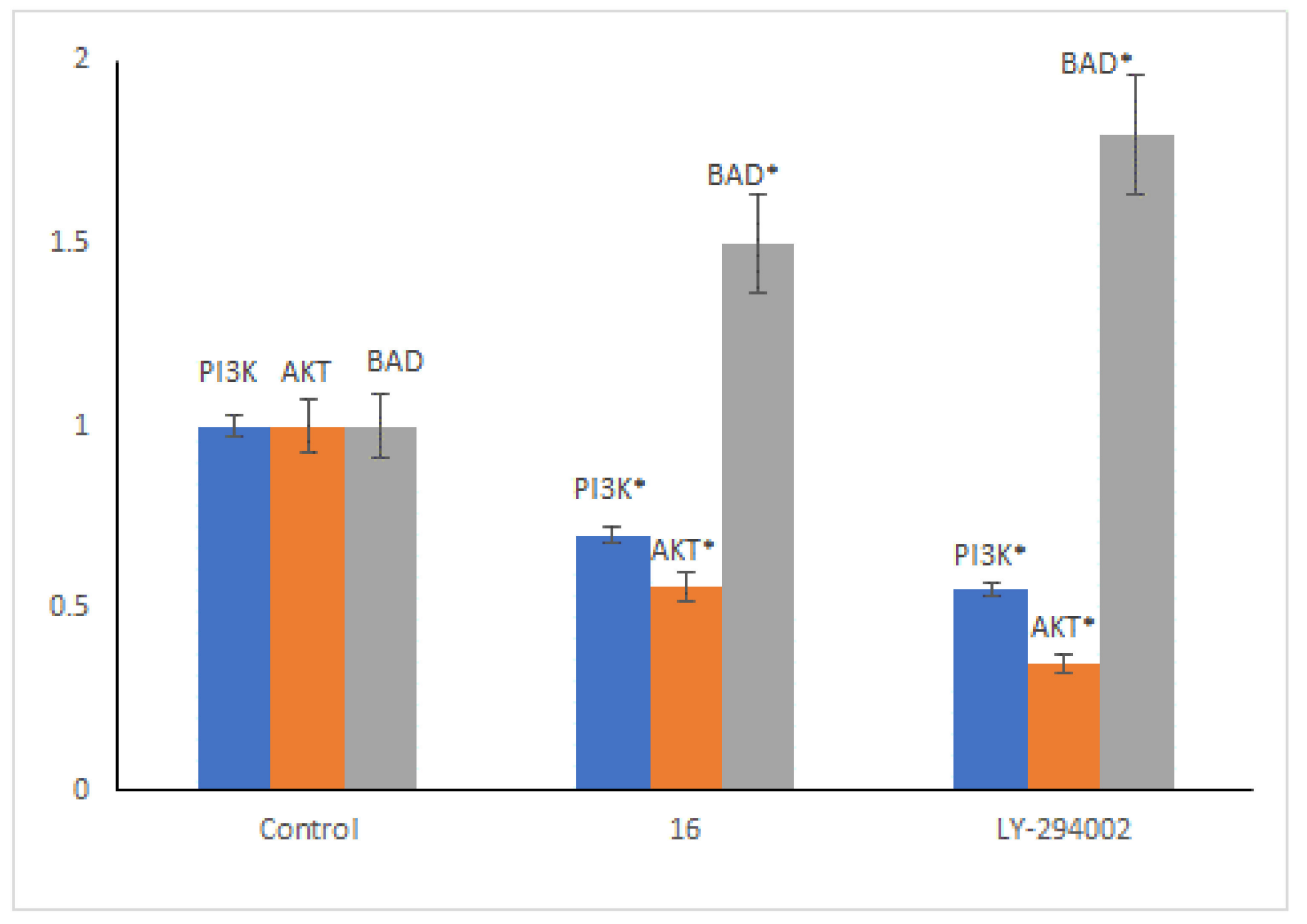
Relative gene expression of PI3K, AKT, and BAD in HCT116 cells after treatment with Compound **16** and the positive control LY-294002. Bars represent mean values ± SEM (lines) of three replicates. Asterisks represent significant change relative to the negative control (* *p* < 0.05).

The effect of compound **16** on the PI3K/AKT signaling pathway was investigated using real time PCR. According to our results, the relative gene expression of PI3K, AKT, and BAD was significantly affected by treatment with compound **16** (1 µM) in a manner consistent with the positive control treatment (1 uM) (Figure 3). A significant decrease in PI3K and AKT gene expression was evident upon treatment with either compound **16** or the positive control LY-294002. On the other hand, the expression of the pro-apoptotic gene BAD was significantly increased in treated cells (for both compound **16** and the positive control) when compared to the negative control where there was no treatment applied.

### 2.3. Computational Studies

#### 2.3.1. Molecular Docking

In order to test whether the anticancer activity of the synthesized compounds (**5**, **7**–**27**) in Caco-2 and HCT-116 cell lines (both strongly upregulate PI3Kα) can be partly attributed to PI3Kα modulation, we performed molecular docking studies in PI3Kα crystal structures. For that, we employed the coordinates of WT (PDB ID: 2RD0) [4] and MUT (H1047R) (PDB ID: 3HHM) [29] PI3Kα to determine the structural basis of the PI3Kα/ligand interaction.

**Figure 4:**
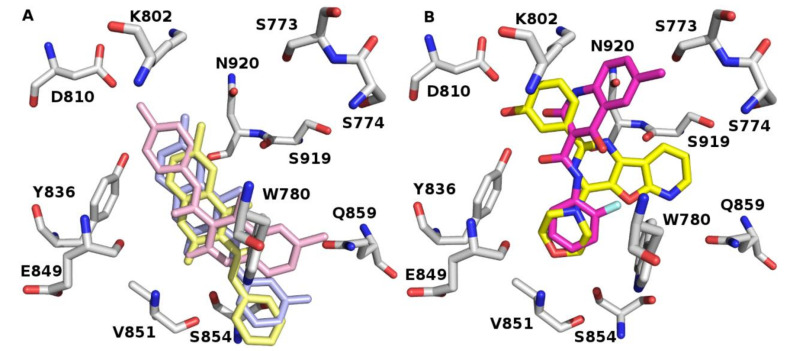
The kinase cleft of (**A**) 2RD0 bears the IF docked poses of some of the synthesized molecules and (**B**) overlaying of the IF docked conformation of **14** (represented in pink color) and the co-crystallized ligand (X6K) (yellow color). Some of key binding residues are disclosed and H atoms are unseen for clarification. Picture visualized by PYMOL [32].

In order to explore the binding interaction of PI3Kα and the verified compounds (**5**, **7**–**27**) in the kinase domain of PI3Kα, we carried out induced-fit docking (IFD) studies [30,31,32] against the kinase domains of 2RD0 and 3HHM. IFD data illustrated that **5**, **7**–**27** reside in PI3Kαs kinase domains and the docked pose of **14** superposes the crystal structure of X6K in the kinase domain of PI3Kα (PDB ID: 4L23) [33] (Figure 4).

**Figure 5:**
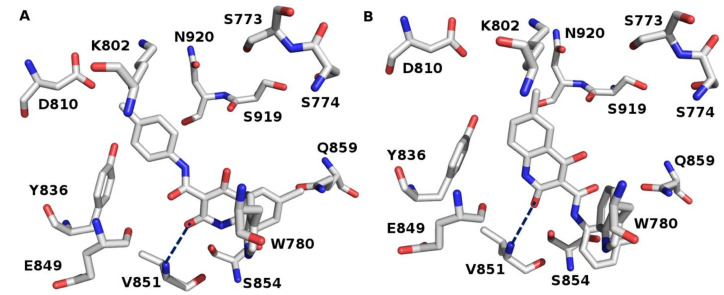
Binding geometries of (**A**) **13** and (**B**) **15** in the kinase site of 2RD0. H-bonds are depicted by blue dotted lines. H atoms are masked for clarification. Picture visualized by PYMOL [32].

The IFD approach probes the conformational changes in proteins in the following manner: ligands are docked to a protein’s binding site recruiting Glide docking and the top ligand geometries are minimized along with protein binding site using the Prime module. Next, a redocking procedure is employed against the relaxed protein. Therefore, protein plasticity is considered during the docking tactic. The backbones of the synthesized molecules form H-bonds with S773, S774, A775, K776, W780, K802, D810, Y836, E849, V851, N853, S854, Q859, D915, H917, S919, N920, and D933 (Table 3, Figure 5). Furthermore, other computational [14,16,17,19,20,34] and experimental studies [4] have assigned the contribution of these key binding amino acids in the PI3Kα/ligand binding interaction. Interestingly, **5**, **7**–**27** displayed comparable affinity toward both WT (2RD0) and MUT (H1047R) (3HHM) PI3Kα. Moreover, the prevalence of S774 in the ligand/PI3Kα interaction suggests that the series might be selective PI3Kα inhibitors [14].

**Figure 6:**
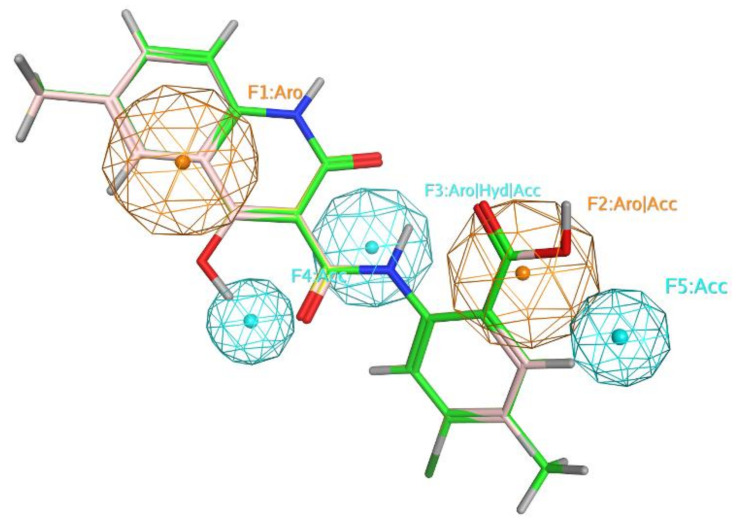
PI3Kα active inhibitors pharmacophore model with **20** (yellow color), **24** (light pink color), and **25** (green color). Picture made by MOE [15].

In order to get further details about the tailored functionalities of **7**–**27**, we screened them against an adopted pharmacophore model of active PI3Kα inhibitors [13]. The backbones of **7**–**27** endorse the fingerprint of active PI3Kα inhibitors (Figure 6), represented as (F1), pointing to one aromatic ring; (F2), one aromatic or H-bond acceptor; (F3), one aromatic or hydrophobic or H-bond acceptor; and (F4) or (F5), one H-bond acceptor. Such a result rationalizes the affinity of the series against PI3Kα. Furthermore, the accommodation of **7**–**27** in the kinase cleft anticipates potential PI3Kα inhibitory activity.

#### 2.3.2. Descriptor Analysis

**Figure 7:**
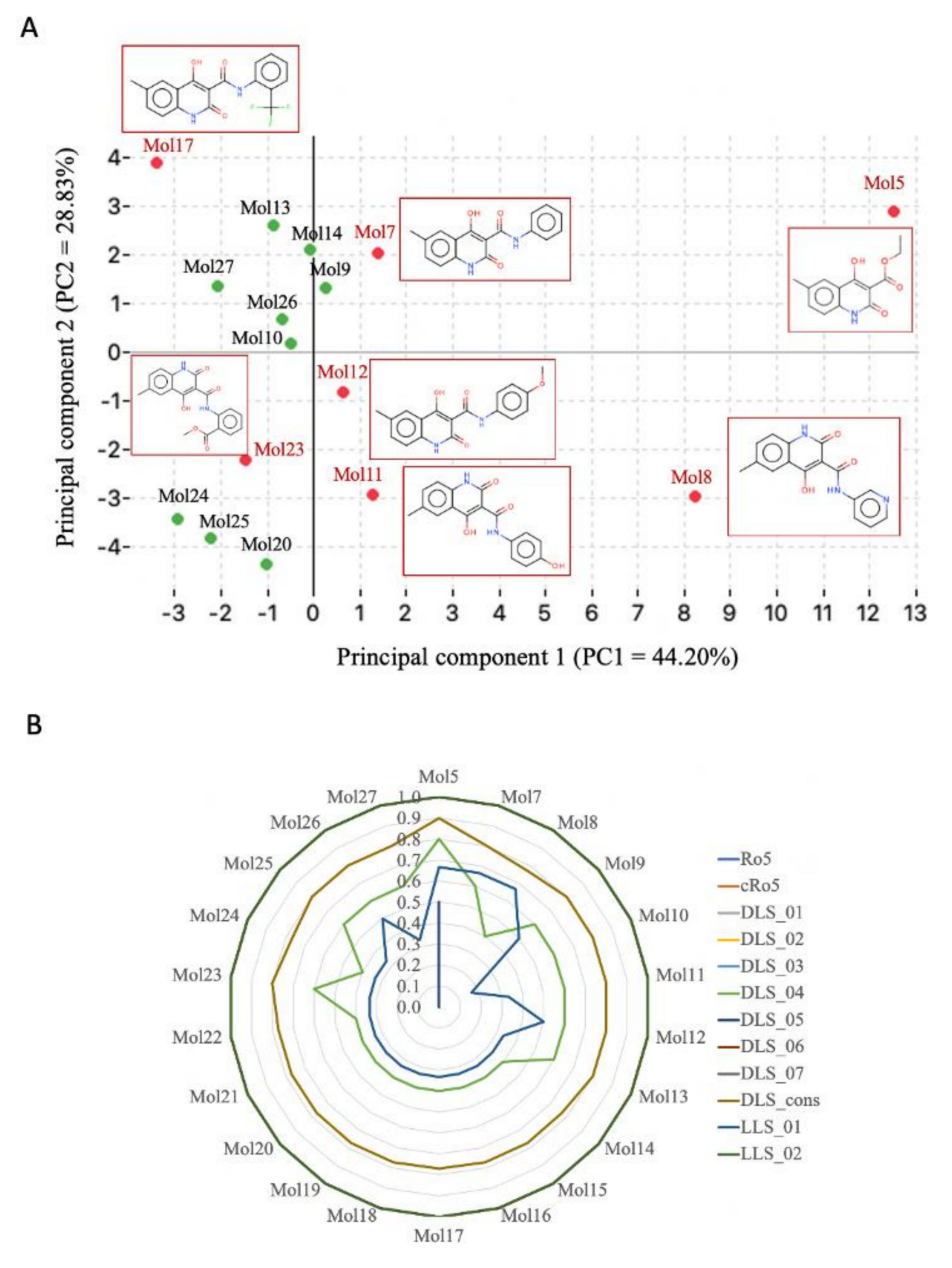
Molecular descriptor analysis. (**A**) A graphical 2D representation of the principal component analysis of molecular descriptors. PC1 explaining 44.20% of the variation in descriptor values is on the X-axis, and PC2 explaining 28.83% of the variation in descriptor values is on the y-axis. The descriptors used for the analysis belong to two major categories of molecular descriptors: (1) drug-like indices; and (2) molecular properties. Structures shown in red boxes, which correspond to the red dots on the figure, serve as structural examples. (**B**) Radar plot of drug-like molecular descriptors. Both drug-like scores (DLS) and lead-like scores (LLS) were calculated for molecules **5** and **7**–**22**. A molecule is drug-like or lead-like if its DLS or LLS is ≥ 0.50.

Molecular descriptors comprising two main categories of [35], drug-like indices and molecular properties, were calculated for molecules **5** and **7**–**27**. All descriptor values were then analyzed using principal component analysis. Our analysis revealed a diversity of lead-like, drug-like, and molecular properties of the synthesized compounds (Figure 7A). All calculated descriptors for all analyzed molecules are reported in Supplementary Materilas Table S1.

Our analysis further revealed that our synthesized molecules differ in their lead-like and drug-like properties as shown in Figure 7B. All synthesized molecules passed the lead-like scoring filter LLS-02 [36], but only molecules **5**, **7** and **8** passed the lead-like scoring filter DDL-01 [37]. Our molecules also showed diverse drug-like properties based on three drug-like scoring filters: DLS-04 [38], DLS-05 [39], and Dragon Consensus Score for drug-likeness [40]. For example, molecule **5** was the only molecule that passed the DLS-05 drug-like filter, while molecules **5**, **7**, **9**–**13**, **23**, **25**–**27** passed the DLS-04 drug-like filter. All values for all calculated drug-like scores are found in Supplementary Materilas Table S1. Drugs that have higher overall drug-like scores have better chances to succeed in further experimental testing including animal studies.

## 3. Materials and Methods

### 3.1. Chemistry

All chemicals, reagents, and solvents were of analytical grade and used directly without further purification. Chemicals were purchased from the corresponding companies: SD Fine-Chem Limited (SDFCL) (Mumbai, India), Acros Organics (Fair Lawn, NJ, USA), Sigma-Aldrich (St. Louis, MO, USA), Fluka (Buchs, Switzerland), Sharlau (Barcelona, Spain), Tedia (Fairfield, OH, USA), and Gainland Chemical Company (GCC) (Sandycroft, Flintshire, UK). Rota vapor (1) model R-215 (Buchi, Switzerland) connected to vacuum pump model v-700 and water bath B-491 were used to evaporate ordinary solvents and vacuum controller v-855. Melting points (MP) were measured using Gallenkamp melting point apparatus (Loughborough, UK). The hot plate and magnetic stirrer were purchased from vision scientific CO, LTD, Westland, MI, USA. Thin layer chromatography (TLC) was performed on 20 × 20 cm, with layer thickness 0.2 mm, aluminum cards pre-coated with fluorescent silica gel GF254 DC (Fluka analytical, Munich, Germany) and visualized by UV light indicator (at 254 and/or 360 nm).

Nuclear magnetic resonance (NMR) ^1^H- and ^13^C-NMR spectra were measured on a Bruker (Mundelein, IL, USA), Avance DPX- 500 MHz spectrophotometer (The University of Jordan) and Bruker NanoBay 400 MHz spectrophotometer (the Hashemite University). Chemical shifts are given in δ (ppm) using TMS as an internal reference; the samples are dissolved in DMSO-d_6_. High-resolution mass spectra (HRMS) were recorded using a Bruker APEX-IV (7 Tesla) instrument. External calibration was executed using an arginine cluster at a mass range of *m*/*z* 175–871, and the samples were dissolved in methanol and drops of formic acid.

### 3.2. Synthesis of Target Compounds

#### 3.2.1. Ethyl 4-hydroxy-6-methyl-2-quinolone-3-carboxylate (**5**)

Sodium ethoxide (6.27 g, 92.16 mmol) was added to a solution of ethyl 2-amino-5-methylbenzoate (**3**) (5.506 g, 30.72, mmol) and diethylmalonate (**4**) (46.86 mL, 307.2 mmol) in DMSO (30 mL). The mixture was refluxed at 130–140 °C for 72 h. Completion of the reaction was achieved by the absence of the compound (**3**) spot on TLC. After cooling, the reaction mixture was acidified with 0.3 M HCl and the precipitate was collected by suction filtration then dried under reduced pressure.

A beige powder (3.35 g) with % yield: 70.2, R_ƒ_ = 0.51 (CHCl_3_: MeOH-9.8: 0.2); m.p: 215–216 °C; ^1^H-NMR (500 Hz, DMSO-*d*_6_): δ = 13.34 (s, 1H, O*H*), 11.38 (s, 1H, N*H*), 7.65 (s, 1H, Ar-*H*), 7.36 (d, *J* = 8.1 Hz, 1H, Ar*-H*), 7.16 (d, *J* = 8.1 Hz, 1H, Ar-*H*), 4.29 (q, *J* = 7.0 Hz, 2H, OC*H*_2_), 2.27 (s, 3H, C*H*_3_), 1.25 (t, *J* = 7.0 Hz, 3H, CH_2_C*H*_3_) ppm; ^13^C-NMR (125 Hz, DMSO-*d*_6_): δ = 170.9 (1C), 169.2 (1C), 159.6 (1C), 138.4 (1C), 135.4 (1C), 131.2 (1C), 123.8 (1C), 115.7 (1C), 113.6 (1C), 100.3 (1C), 61.8 (1C), 20.9 (1C), 14.5 (1C) ppm.

#### 3.2.2. *N*-Phenyl-4-hydroxy-6-methyl-2-quinolone-3-carboxamide (**7**)

Ethyl 4-hydroxy-6-methyl-2-quinolone-3-carboxylate (**5**) (1 g, 4.044 mmol) and aniline (**6a**) (1.13 g, 12.13 mmol) were dissolved in 25 mL THF. A few drops of DMF were added, then the mixture was refluxed at 150–160 °C for 48 h. Completion of the reaction was indicated by absence of **5** on TLC and appearance of precipitate at RT. The solid was isolated by suction filtration, washed with absolute ethanol, and dried under reduced pressure.

A beige powder (0.67 g) with %yield: 56, R_ƒ_ = 0.68 (CHCl_3_: MeOH-9.7: 0.3); m.p: 302–304 °C; ^1^H-NMR (500 Hz, DMSO-d_6_ + NaOD): δ = 7.72 (s, 1H, Ar-H), 7.64 (d, J = 8.0 Hz, 2H, Ar-H), 7.17 (t, J = 8.0 Hz, 2H, Ar-H), 7.05 (d, J = 8 Hz, 1H, Ar-H), 6.94 (d, J = 8.0 Hz, 1H, Ar-H), 6.87 (t, J = 7.0 Hz, 1H, Ar-H), 2.31 (s, 3H, CH_3_) ppm;^13^C-NMR (125 Hz, DMSO-d_6_+NaOD): 177.4 (1C), 168.8 (1C), 168.4 (1C), 141.2 (1C), 131.8 (1C), 129.0 (3C), 127.5 (1C), 125.5 (1C), 122.2 (1C), 121.9 (1C), 119.9 (2C), 117.6 (1C), 99.8 (1C), 21.2 (1C) ppm. High-resolution mass spectrum, *m/z*: calculated 295.10827 for C_17_H_15_N_2_O_3_ [M + H]^+^; found 295.10804.

#### 3.2.3. *N*-(Pyridin-3-yl)-4-hydroxy-6-methyl-2-quinolone-3-carboxamide (**8**)

Ethyl 4-hydroxy-6-methyl-2-quinolone-3-carboxylate (**5**) (1 g, 4.044 mmol) and 3-aminopyridine (**6b**) (1.13 g, 12.03 mmol) were dissolved in 25 mL THF. A few drops of DMF were added then the mixture was refluxed at 150–160 °C for 48 h. Completion of the reaction was indicated by absence of **5** on TLC and appearance of precipitate at RT. The solid was isolated by filtration, washed with absolute ethanol, and dried under reduced pressure.

A beige powder (0.96 g) with %yield: 80, R_ƒ_ = 0.6 (CHCl_3_: MeOH-9.5: 0.5); m.p: 296–298 °C; ^1^H-NMR (500 Hz, DMSO-*d*_6_ +NaOD): δ = 8.81 (s, 1H, Ar-*H*), 8.21 (d, *J* = 6.7 Hz, 1H, Ar-*H*), 8.07 (d, *J* = 6.7 Hz, 1H, Ar-*H*), 7.69 (s, 1H, Ar-*H*), 7.23 (t, *J* = 7 Hz, 1H, Ar-*H*), 6.97 (d, *J =* 7.2 Hz, 1H, Ar-*H*), 6.93 (d, *J =* 7.2 Hz, 1H, Ar-*H*), 2.26 (s, 3H, C*H*_3_) ppm; ^13^C-NMR (125 Hz, DMSO-*d*_6_+NaOD): δ = 177.7 (1C), 173.1 (1C), 170.8 (1C), 148.2 (1C), 141.9 (1C), 141.4 (1C), 138.6 (1C), 130.8 (1C), 126.5 (1C), 125.0 (1C), 124.4 (1C), 124.0 (1C), 122.9 (1C), 100.6 (1C), 21.1 (1C) ppm. High-resolution mass spectrum, *m/z*: calculated 296.10352 for C_16_H_14_N_3_O_3_ [M + H]^+^; found 296.10341.

#### 3.2.4. *N*-Benzyl-4-hydroxy-6-methyl-2-quinolone-3-carboxamide (**9**)

Ethyl 4-hydroxy-6-methyl-2-quinolone-3-carboxylate (**5**) (0.95 g, 3.84 mmol) and benzylamine (**6c**) (1.23 g, 11.52 mmol) were dissolved in 25 mL THF. A few drops of DMF were added then the mixture was refluxed at 150–160 °C for 48 h. Completion of the reaction was indicated by absence of **5** on TLC and appearance of precipitate at RT. The solid was isolated by suction filtration, washed with absolute ethanol, and dried under reduced pressure.

A white powder (0.87 g) with %yield: 74, R_ƒ_ = 0.75 (CHCl_3_: MeOH-9.7: 0.3); m.p: 233–235 °C; ^1^H-NMR (500 Hz, DMSO-*d*_6_): δ = 17.01 (s, 1H, O*H*), 11.78 (s, 1H, N*H*), 10.71 (s, 1H, N*H*), 7.75 (s,1H, Ar-*H*), 7.37 (m, 7H, Ar-*H*), 4.6 (s, 2H, C*H*_2_), 2.38 (s, 3H, C*H*_3_) ppm; ^13^C-NMR (125 Hz, DMSO-*d*_6_): δ = 172.5 (1C), 171.2 (1C), 162.9 (1C), 138.7 (1C), 137.4 (1C), 135.6 (1C), 132.0 (1C), 129.0 (2C), 128.0 (2C), 127.7 (1C), 123.6 (1C), 116.3 (1C), 114.6 (1C), 96.6 (1C), 42.6 (1C), 20.9 (1C) ppm. High-resolution mass spectrum, *m/z*: calculated 309.12392 for C_18_H_17_N_2_O_3_ [M + H]^+^; found 309.12345.

#### 3.2.5. *N*-(Anilino)-4-hydroxy-6-methyl-2-quinolone-3-carboxamide (**10**)

Ethyl 4-hydroxy-6-methyl-2-quinolone-3-carboxylate (**5**) (0.95 g, 3.82 mmol) and phenyl hydrazine (**6d**) (1.23 g, 11.38 mmol) were dissolved in 25 mL THF. A few drops of DMF were added and the mixture was refluxed at 150–160 °C for 72 h. Completion of the reaction was indicated by absence of **5** on TLC and appearance of precipitate at RT. The solid was isolated by filtration, washed with absolute ethanol, and dried under reduced pressure.

A beige powder (0.67 g) with %yield: 57, R_ƒ_ = 0.475 (CHCl_3_: MeOH-9.7: 0.3); m.p: 245–247 °C; ^1^H-NMR (500 Hz, DMSO-*d*_6_): δ = 16.3 (s, 1H, O*H*), 11.93 (s, 1H, CON*H*), 11.58 (s, 1H, CON*H*), 8.17 (s, 1H, HNN*H*), 7.74 (s, 1H, Ar-*H*), 7.51 (d, J = 4.6 Hz, 1H, Ar-*H*), 7.29 (d, *J* = 4.6 Hz, 1H, Ar-*H*), 7.20 (m, 2H, Ar-*H*), 6.80 (m, 3H, Ar-*H*), 2.37 (s, 3H, C*H*_3_); ^13^C-NMR (125 Hz, DMSO-*d*_6_): δ = 172.3 (1C), 171.6 (1C), 162.7 (1C), 148.7 (1C), 137.4 (1C), 135.9 (1C), 132.2 (1C), 129.4 (2C), 123.6 (1C), 119.7 (1C), 116.3 (1C), 114.3 (1C), 112.8 (2C), 96.6 (1C), 20.9 (1C) ppm. High-resolution mass spectrum, *m/z*: calculated 310.11917 for C_17_H_16_N_3_O_3_ [M + H]^+^; found 310.11895.

#### 3.2.6. *N*-(4-hydroxyphenyl) 4-hydroxy-6-methyl-2-quinolone-3-carboxamide (**11**)

Ethyl 4-hydroxy-6-methyl-2-quinolone-3-carboxylate carboxylate (**5**) (1 g, 4.044 mmol) and 4-aminophenol (**6e**) (1.32 g, 12.12 mmol) were dissolved in 25 mL THF. A few drops of DMF were added then the mixture was heated at 150 °C for 48 h. Completion of the reaction was indicated by absence of **5** on TLC and appearance of precipitate at RT. The solid was isolated by filtration, washed with absolute ethanol, and dried under reduced pressure.

An off-white powder (0.78 g) with %yield: 62, R_ƒ_ = 0.6 (CHCl_3_:MeOH-9.5: 0.5); m.p: 298–300 °C; ^1^H-NMR (500 Hz, DMSO-*d*_6_): δ = 16.64 (s, 1H, O*H*), 12.41 (s,1H, N*H*), 11.87 (s,1H, N*H*), 9.43 (s, 1H, Ar-O*H*), 7.73 (s, 1H, Ar-*H*), 7.48 (d, *J* = 8.0 Hz, 1H, Ar-*H*), 7.43 (d, *J* = 8.4 Hz, 2H, Ar-*H*), 7.29 (d, *J* = 8.0 Hz, 1H, Ar-*H*), 6.8 (d, *J* = 8.4 Hz, 2H, Ar-*H*), 2.36 (s, 3H, C*H*_3_) ppm; ^13^C-NMR (125 Hz, DMSO-*d*_6_): δ = 172.4 (1C), 168.8 (1C), 163.1 (1C), 155.1 (1C), 137.1 (1C), 135.7 (1C), 132.2 (1C), 128.8 (1C), 123.5 (1C), 122.9 (2C), 116.3 (1C), 116.0 (2C), 114.6 (1C), 96.8 (1C), 20.9 (1C) ppm. High-resolution mass spectrum, *m/z*: calculated 311.10318 for C_17_H_15_N_2_O_4_ [M + H]^+^; found 311.10345.

#### 3.2.7. *N*-(4-methoxyphenyl)-4-hydroxy-6-methyl-2-quinolone-3-carboxamide (**12**)

Ethyl 4-hydroxy-6-methyl-2-quinolone-3-carboxylate (**5**) (0.95 g, 3.82 mmol) and *p*-anisidine (**6f**) (0.95 g, 7.64 mmol) were dissolved in 25 mL THF. A few drops of DMF were added then the mixture was refluxed at 150–176 °C for 48 h. Completion of the reaction was indicated by absence of **5** on TLC and appearance of precipitate at RT. The solid was isolated by suction filtration, washed with absolute ethanol, and dried under reduced pressure.

An off-white powder (1.09 g) with %yield: 88, R_ƒ_ = 0.65 (CHCl_3_: MeOH-9.7: 0.3); m.p: 275–277 °C; ^1^H-NMR (500 Hz, DMSO-*d*_6_): δ = 16.58 (s,1H, O*H*), 12.52 (s, 1H, N*H*), 11.95 (s, 1H, N*H*), 7.81 (s, 1H, Ar-*H*), 7.57 (d, *J=* 8.0 Hz, 3H, Ar-*H*), 7.34 (d, *J* = 8.0 Hz, 1H, Ar-*H*), 6.99 (d, *J* = 8.0 Hz, 2H, Ar-*H*), 3.78 (s, 3H, OC*H*_3_), 2.40 (s, 3H, C*H*_3_) ppm; ^13^C-NMR (125 Hz, DMSO-*d*6): δ = 177.1 (1C), 173.1 (1C), 170.2 (1C), 154.2 (1C), 147.6 (1C), 135.1 (1C), 130.7 (1C), 125.0 (1C), 124.6 (1C), 122.9 (1C), 122.3 (1C), 121.4 (2C), 114.1 (2C), 100.9 (1C), 55.5 (1C), 21.4 (1C) ppm. High-resolution mass spectrum, *m/z*: calculated 325.11883 for C_18_H_17_N_2_O_4_ [M + H]^+^; found 325.11825.

#### 3.2.8. *N*-(p-tolyl)-4-hydroxy-6-methyl-2-quinolone-3-carboxamide (**13**)

Ethyl 4-hydroxy-6-methyl-2-quinolone-3-carboxylate (**5**) (1 g, 4.044 mmol) and *p*-toluidine (1.2 g, 11.20 mmol) (**6g**) were dissolved in 25 mL THF. A few drops of DMF was added then the mixture was refluxed at 150–160 °C for 48 h. Completion of the reaction was indicated by absence of **5** on TLC and appearance of precipitate at RT. The solid was isolated by filtration, washed with absolute ethanol, and dried under reduced pressure.

A light yellow powder (0.73 g) with %yield: 71, R_ƒ_ = 0.65 (CHCl_3_: MeOH-9.8: 0.2); m.p: 298–300 °C; ^1^H-NMR (500 Hz, DMSO-*d*_6_ +NaOD): δ = 7.66 (s, 1H, Ar-*H*), 7.54 (d, *J* = 7.7 Hz, 2H, Ar-*H*), 6.96-6.88 (m, 4H, Ar-*H*), 2.22 (s, 3H, C*H*_3_), 2.17 (s, 3H, C*H*_3_) ppm; ^13^C-NMR (125 Hz, DMSO-*d*_6_+NaOD): δ = 177.1 (1C), 172.5 (1C), 170.1 (1C), 146.9 (1C), 139.0 (1C), 130.9 (1C), 130.2 (1C), 129.4 (2C), 125.1 (1C), 125.0 (1C), 122.7 (1C), 121.8 (1C), 120.1 (2C), 100.93 (1C), 21.3 (1C), 20.9 (1C) ppm. High-resolution mass spectrum, *m/z*: calculated 309.12392 for C_18_H_17_N_2_O_3_ [M + H]^+^; found 309.12289.

#### 3.2.9. *N*-(2-fluorophenyl)-4-hydroxy-6-methyl-2-quinolone-3-carboxamide (**14**)

Ethyl 4-hydroxy-6-methyl-2-quinolone-3-carboxylate (**5**) (1 g, 4.044 mmol) and 2-fluoroaniline (**6h**) (1.20 g, 11.20 mmol) were dissolved in 25 mL THF. A few drops of DMF was added then the mixture was refluxed at 150–160 °C for 48 h. Completion of the reaction was indicated by **5** on TLC and appearance of precipitate at RT. The solid was isolated by filtration, washed with absolute ethanol, and dried under reduced pressure.

A beige powder (0.51 g) with %yield: 41, R_ƒ_ = 0.66 (CHCl_3_: MeOH-9.8: 0.2); m.p: 296–298 °C; ^1^H-NMR (400 Hz, DMSO-*d*_6_ +NaOD): δ = 8.75-8.65 (m, 1H, Ar-*H*), 7.72 (s, 1H, Ar-*H*), 7.16–7.02 (m, 1H, Ar-*H*), 7.0–6.94 (m, 2H, Ar-*H*), 6.93-6.90 (m, 2H, Ar-*H*), 2.27 (s, 3H, C*H*_3_) ppm; ^13^C-NMR (100 Hz, DMSO-*d*_6_+NaOD): δ = 177.7 (1C), 174.8 (1C), 170.9 (1C), 169.8 (1C), 153.7(1C), 151.3 (1C), 144.9 (1C), 138.1 (1C), 130.1 (1C), 129.9 (1C), 124.6 (1C), 123.3 (1C), 122.1 (1C), 121.2 (1C), 114.8 (1C), 100.2 (1C), 21.3 (1C) ppm. High-resolution mass spectrum, *m/z*: calculated 313.09885 for C_17_H_14_FN_2_O_3_ [M + H]^+^; found 313.09824.

#### 3.2.10. *N*-(3-fluorophenyl)-4-hydroxy-6-methyl-2-quinolone-3-carboxamide (**15**)

Ethyl 4-hydroxy-6-methyl-2-quinolone-3-carboxylate (**5**) (1 g, 4.044 mmol) and 3-fluoroaniline (**6i**) (1.20 g, 11.20 mmol) were dissolved in 25 mL THF. A few drops of DMF was added then the mixture was refluxed at 150–160 °C for 48 h. Completion of the reaction was indicated by absence of **5** on TLC and appearance of precipitate at RT. The solid was isolated by filtration, washed with absolute ethanol, and dried under reduced pressure.

A beige powder (0.58 g) with %yield: 46, R_ƒ_ = 0.70 (CHCl_3_: MeOH-9.8: 0.2); m.p: 294–296 °C; ^1^H-NMR (500 Hz, DMSO-*d*_6_ +NaOD): δ = 7.89–7.87 (m, 1H, Ar-*H*), 7.63 (s, 1H, Ar-*H*), 7.21–7.11 (m, 2H, Ar-*H*), 6.92 (d, *J* = 8.7 Hz, 1H, Ar-*H*), 6.88 (d, *J =* 8 Hz, 1H, Ar-*H*), 6.65-6.55 (m, 1H, Ar-*H*), 2.20 (s, 3H, C*H*_3_) ppm; ^13^C-NMR (125 Hz, DMSO-*d*_6_+NaOD): δ = 177.5 (1C), 172.8 (1C), 170.4 (1C), 163.9 (1C), 161.9 (1C), 147.9 (1C), 143.7 (1C), 143.6 (1C), 130.2 (1C), 130.1 (1C), 124.9 (1C), 122.7 (1C), 115.5 (1C), 107.5 (1C), 106.6 (1C), 100.8 (1C), 21.3 (1C) ppm. High-resolution mass spectrum, *m/z*: calculated 313.09885 for C_17_H_14_FN_2_O_3_ [M + H]^+^; found 313.09879.

#### 3.2.11. *N*-(4-fluorophenyl)-4-hydroxy-6-methyl-2-quinolone-3-carboxamide (**16**)

Ethyl 4-hydroxy-6-methyl-2-quinolone-3-carboxylate (**5**) (1 g, 4.044 mmol) and 2-fluoroaniline (**6j**) (1.20 g, 11.20 mmol) were dissolved in 25 mL THF. A few drops of DMF was added then the mixture was refluxed at 150–160 °C for 48 h. Completion of the reaction was indicated by absence of **5** on TLC and appearance of precipitate at RT. The solid was isolated by filtration, washed with absolute ethanol, and dried under reduced pressure.

A beige powder (0.71 g) with %yield: 56, R_ƒ_ = 0.61 (CHCl_3_: MeOH-9.8: 0.2); m.p: 303–305 °C; ^1^H-NMR (400 Hz, DMSO-*d*_6_ +NaOD): δ = 7.71–7.69 (m, 3H, Ar-*H*), 7.02–6.96 (m, 3H, Ar-*H*), 6.91 (s, 1H, Ar-*H*), 2.27 (s, 3H, C*H*_3_) ppm; ^13^C-NMR (100 Hz, DMSO-*d*_6_+NaOD): δ = 177.3 (1C), 172.4 (1C), 170.0 (1C), 158.3 (1C), 155.9 (1C), 147.2 (1C), 143.9 (1C), 138.3 (1C), 130.7 (1C), 125.0 (1C), 124.5 (1C), 122.8 (2C), 115.3 (1C), 115.09 (1C), 100.5 (1C), 21.3 (1C) ppm. High-resolution mass spectrum, *m/z*: calculated 313.09885 for C_17_H_14_FN_2_O_3_ [M + H]^+^; found 313.09758.

#### 3.2.12. *N*-(2-(trifluoromethyl)phenyl)-4-hydroxy-6-methyl-2-quinolone-3-carboxamide (**17**)

Ethyl 4-hydroxy-6-methyl-2-quinolone-3-carboxylate (**5**) (1 g, 4.044 mmol) and 2-trifluromethyl aniline (**6k**) (1.8 g, 11.20 mmol) were dissolved in 25 mL THF. A few drops of DMF was added then the mixture was refluxed at 150–160 °C for 48 h. Completion of the reaction was indicated by absence of **5** on TLC and appearance of precipitate at RT. The solid was isolated by filtration, washed with absolute ethanol, and dried under reduced pressure.

A white powder (0.89 g) with %yield: 67, R_ƒ_ = 0.64 (CHCl_3_: MeOH-9.8: 0.2); m.p: 285–287 °C; ^1^H-NMR (400 Hz, DMSO-*d*_6_ +NaOD): δ = 8.43 (d, *J* = 8.4 Hz, 1H, Ar-*H*), 7.68 (s, 1H, Ar-*H*), 7.57 (d, *J* = 8.0 Hz, 1H, Ar-*H*), 7.43 (t, *J* = 7.6 Hz, 1H, Ar-*H*), 7.07 (t, *J* = 8.0 Hz, 1H, Ar-*H*), 6.99-6.97 (m, 1H, Ar-*H*), 6.91-6.89 (m, 1H, Ar-*H*), 2.27 (s, 3H, C*H*_3_) ppm; ^13^C-NMR (100 Hz, DMSO-*d*_6_+NaOD): δ = 178.0 (1C), 172.5 (1C), 170.2 (1C), 139.7 (1C), 132.4 (1C), 130.8 (1C), 128.9 (1C), 125.2 (1C), 124.3 (1C), 123.4 (1C), 122.7 (1C), 121.7 (1C), 121.4 (1C), 120.7 (1C), 119.2 (1C), 118.9 (1C), 100.0 (1C), 21.3 (1C) ppm. High-resolution mass spectrum, *m*/*z*: calculated 363.09565 for C_18_H_14_F_3_N_2_O_3_ [M + H]^+^; found 363.09516.

#### 3.2.13. *N*-(3-(trifluoromethyl)phenyl)-4-hydroxy-6-methyl-2-quinolone-3-carboxamide (**18**)

Ethyl 4-hydroxy-6-methyl-2-quinolone-3-carboxylate (**5**) (1 g, 4.044 mmol) and 3-trifluromethyl aniline (**6l**) (1.8 g, 11.20 mmol) were dissolved in 25 mL THF. A few drops of DMF was added then the mixture was refluxed at 150–160 °C for 48 h. Completion of the reaction was indicated by absence of **5** on TLC and appearance of precipitate at RT. The solid was isolated by filtration, washed with absolute ethanol, and dried under reduced pressure.

A white powder (0.95 g) with %yield: 75, R_ƒ_ = 0.60 (CHCl_3_: MeOH-9.8: 0.2); m.p: 302–304 °C; ^1^H-NMR (400 Hz, DMSO-*d*_6_ +NaOD): δ = 8.25 (s, 1H, Ar-*H*), 7.76 (d, *J* = 7.6 Hz, 1H, Ar-*H*), 7.70 (s, 1H, Ar-*H*), 7.43 (t, *J* =8.0 Hz, 1H, Ar-*H*), 7.23–7.15 (m, 1H, Ar-*H*), 6.99–6.89 (m, 2H, Ar-*H*), 2.26 (s, 3H, C*H*_3_) ppm; ^13^C-NMR (100 Hz, DMSO-*d*_6_+NaOD): δ = 177.6 (1C), 170.4 (1C), 170.0 (1C), 129.9 (1C), 125.1 (1C), 125.0 (3C), 123.5 (1C), 123.1 (1C), 122.9 (3C), 122.7 (1C), 120.9 (2C), 100.5 (1C), 21.3 (1C) ppm. High-resolution mass spectrum, *m/z*: calculated 363.09565 for C_18_H_14_F_3_N_2_O_3_ [M + H]^+^; found 363.09524.

#### 3.2.14. *N*-(4-(trifluoromethyl)phenyl)-4-hydroxy-6-methyl-2-quinolone-3-carboxamide (**19**)

Ethyl 4-hydroxy-6-methyl-2-quinolone-3-carboxylate (**5**) (1 g, 4.044 mmol) and 4-trifluromethyl aniline (**6m**) (1.8 g, 11.20 mmol) were dissolved in 25 mL THF. A few drops of DMF was added then the mixture was refluxed at 150–160 °C for 48 h. Completion of the reaction was indicated by absence of **5** on TLC and appearance of precipitate at RT. The solid was isolated by filtration, washed with absolute ethanol, and dried under reduced pressure.

A white powder (1.08 g) with %yield: 85, R_ƒ_ = 0.59 (CHCl_3_: MeOH-9.8: 0.2); m.p: 317–319 °C; ^1^H-NMR (400 Hz, DMSO-*d*_6_ +NaOD): δ = 7.88 (d, *J* = 8.8 Hz, 2H, Ar-*H*), 7.53 (s, 1H, Ar-*H*), 7.51 (d, *J* = 8.4 Hz, 2H, Ar-*H*), 7.09–6.79 (m, 2H, Ar-*H*), 2.29 (s, 3H, C*H*_3_) ppm; ^13^C-NMR (100 Hz, DMSO-*d*_6_+NaOD): δ = 177.6 (1C), 170.4 (1C), 170.0 (1C), 129.9 (1C), 125.1 (1C), 125.0 (3C), 123.5 (1C), 123.1 (1C), 122.9 (3C), 122.7 (1C), 120.9 (2C), 100.5 (1C), 21.3 (1C) ppm. High-resolution mass spectrum, *m/z*: calculated 363.09565 for C_18_H_14_F_3_N_2_O_3_ [M + H]^+^; found 363.09587.

#### 3.2.15. *N*-(2-benzoic acid)-4-hydroxy-6-methyl-2-quinolone-3-carboxamide (**20**)

Ethyl 4-hydroxy-6-methyl-2-quinolone-3-carboxylate (**5**) (1 g, 4.044 mmol) and 2-amino benzoic acid (**6n**) (1.50 g, 11.20 mmol) were dissolved in 25 mL THF. A few drops of DMF was added then the mixture was refluxed at 150–160 °C for 48 h. Completion of the reaction was indicated by absence of **5** on TLC and appearance of precipitate at RT. The solid was isolated by filtration, washed with absolute ethanol, and dried under reduced pressure.

A white powder (0.93 g) with %yield: 80, R_ƒ_ = 0.53 (CHCl_3_: MeOH-9.0: 1.0); m.p: 290–292 °C; ^1^H-NMR (400 Hz, DMSO-d_6_ +NaOD): δ = 8.60 (d, J = 8.4 Hz, 1H, Ar-H), 7.79–7.69 (m, 2H, Ar-H), 7.11 (t, J = 7.6 Hz, 1H, Ar-H), 6.95–6.89 (m, 2H, Ar-H), 6.82–6.79 (m, 1H, Ar-H), 2.23 (s, 3H, CH_3_) ppm; ^13^C-NMR (100 Hz, DMSO-d_6_+NaOD): δ = 176.8 (2C), 172.9 (1C), 170.2 (1C), 146.9 (1C), 140.1 (1C), 130.7 (1C), 130.4 (1C), 130.1 (1C), 128.4 (1C), 125.4 (1C), 125.0 (1C), 124.6 (1C), 124.5 (1C), 123.7 (1C), 123.5 (1C), 102.1 (1C), 21.3 (1C) ppm. High-resolution mass spectrum, m/z: calculated 339.09810 for C_18_H_15_N_2_O_5_ [M + H]^+^; found 339.09898.

#### 3.2.16. *N*-(3-benzoic acid)-4-hydroxy-6-methyl-2-quinolone-3-carboxamide (**21**)

Ethyl 4-hydroxy-6-methyl-2-quinolone-3-carboxylate (**5**) (1 g, 4.044 mmol) and 3-amino benzoic acid (**6o**) (1.50 g, 11.20 mmol) were dissolved in 25 mL THF. A few drops of DMF was added then the mixture was refluxed at 150–160 °C for 48 h. Completion of the reaction was indicated by absence of **5** on TLC and appearance of precipitate at RT. The solid was isolated by filtration, washed with absolute ethanol, and dried under reduced pressure.

A beige powder (1.03 g) with %yield: 88, R_ƒ_ = 0.34 (CHCl_3_: MeOH-9.5: 0.5); m.p: 303–305 °C; ^1^H-NMR (400 Hz, DMSO-d_6_ +NaOD): δ = 8.17 (s, 1H, Ar-H), 7.74 (d, J = 7.6 Hz, 1H, Ar-H), 7.69 (s, 1H, Ar-H), 7.45 (d, J = 7.6 Hz, 1H, Ar-H), 7.14 (t, J = 7.6 Hz, 1H, Ar-H), 6.98–6.89 (m, 2H, Ar-H), 2.27 (s, 3H, CH_3_) ppm; ^13^C-NMR (100 Hz, DMSO-d_6_+NaOD): δ = 177.2 (1C), 173.1 (1C), 171.5 (1C), 170.3 (1C), 147.9 (1C), 140.9 (1C), 140.2 (1C), 127.6 (1C), 125.0 (1C), 124.0 (1C), 122.9 (1C), 122.5 (1C), 122.3 (1C), 121.4 (1C), 120.8 (1C), 100.9 (1C), 21.4 (1C) ppm. High-resolution mass spectrum, m/z: calculated 339.09810 for C_18_H_15_N_2_O_5_ [M + H]^+^; found 339.09854.

#### 3.2.17. *N*-(4-benzoic acid)-4-hydroxy-6-methyl-2-quinolone-3-carboxamide (**22**)

Ethyl 4-hydroxy-6-methyl-2-quinolone-3-carboxylate (**5**) (1 g, 4.044 mmol) and 4-amino benzoic acid (**6p**) (1.50 g, 11.20 mmol) were dissolved in 25 mL THF. A few drops of DMF was added then the mixture was refluxed at 150–160 °C for 48 h. Completion of the reaction was indicated by absence of **5** on TLC and appearance of precipitate at RT. The solid was isolated by filtration, washed with absolute ethanol, and dried under reduced pressure.

A yellow powder (0.99 g) with %yield: 83, R_ƒ_ = 0.2 (CHCl_3_: MeOH-9.5: 0.5); m.p: 330–332 °C; ^1^H-NMR (500 Hz, DMSO-d_6_ +NaOD): δ = 7.79 (s, 1H, Ar-H), 7.64–7.54 (m, 2H, Ar-H), 6.93 (d, J = 8 Hz, 2H, Ar-H), 6.87 (d, J = 8 Hz, 2H, Ar-H), 2.23 (s, 3H, CH_3_) ppm; ^13^C-NMR (125 Hz, DMSO-d_6_+NaOD): δ = 177.3 (1C), 172.9 (1C), 171.7 (1C), 170.3 (1C), 147.8 (1C), 142.9 (1C), 132.3 (1C), 130.8 (1C), 130.3 (2C), 124.9 (1C), 124.5 (1C), 122.8 (1C), 122.2 (1C), 118.4 (2C), 100.9 (1C), 21.3 (1C) ppm. High-resolution mass spectrum, m/z: calculated 339.09810 for C_18_H_15_N_2_O_5_ [M + H]^+^; found 339.09817.

#### 3.2.18. *N*-(2-methyl benzoate)-4-hydroxy-6-methyl-2-quinolone-3-carboxamide (**23**)

Ethyl 4-hydroxy-6-methyl-2-quinolone-3-carboxylate (**5**) (1 g, 4.044 mmol) and methyl 2-amino benzoate (**6q**) (1.69 g, 11.20 mmol) were dissolved in 25 mL THF. A few drops of DMF was added then the mixture was refluxed at 150–160 °C for 48 h. Completion of the reaction was indicated by absence of **5** on TLC and appearance of precipitate at RT. The solid was isolated by filtration, washed with absolute ethanol, and dried under reduced pressure.

A white powder (1.2 g) with %yield: 93, R_ƒ_ = 0.65 (CHCl_3_: MeOH-9.8:0.2); m.p: 237–239 °C; ^1^H-NMR (400 Hz, DMSO-d_6_ +NaOD): δ = 8.58 (d, J = 8.0 Hz, 1H, Ar-H), 7.78 (s, 1H, Ar-H), 7.66 (d, J = 8.0 Hz, 1H, Ar-H), 7.10 (t, J = 7.2 Hz, 1H, Ar-H), 6.96-6.89 (m, 2H, Ar-H), 6.83 (t, J = 7.6 Hz, 1H, Ar-H), 3.7 (s, 3H, -COOCH_3_), 2.23 (s, 3H, CH_3_) ppm; ^13^C-NMR (100 Hz, DMSO-d_6_+NaOD): δ = 172.9 (1C), 172.8 (1C), 171.9 (1C), 170.4 (1C), 146.4 (1C), 140.9 (1C), 130.7 (1C), 130.4 (2C), 128.4 (2C), 125.3 (1C), 124.2 (1C), 123.6 (1C), 122.2 (1C), 121.9 (1C), 102.1 (1C), 56.4 (1C), 21.3 (1C) ppm. High-resolution mass spectrum, m/z: calculated 353.11375 for C_19_H_17_N_2_O_5_ [M + H]^+^; found 353.11375.

#### 3.2.19. *N*-(4-chlorobenzoic acid)-4-hydroxy-6-methyl-2-quinolone-3-carboxamide (**24**)

Ethyl 4-hydroxy-6-methyl-2-quinolone-3-carboxylate (**5**) (1 g, 4.044 mmol) and 2-amino-4- chloro- benzoic acid (**6r**) (1.90 g, 11.20 mmol) were dissolved in 25 mL THF. A few drops of DMF was added then the mixture was refluxed at 150–160 °C for 48 h. Completion of the reaction was indicated by absence of **5** on TLC and appearance of precipitate at RT. The solid was isolated by filtration, washed with absolute ethanol, and dried under reduced pressure.

A white powder (0.15 g) with %yield: 10, R_ƒ_ = 0.21 (CHCl_3_: MeOH-8.0: 2.0); m.p: 286–289 °C; ^1^H-NMR (400 Hz, DMSO-*d*_6_ +NaOD): δ = 8.83 (s, 1H, Ar-*H*), 7.79-7.72 (m, 2H, Ar-*H*), 6.93–6.85 (m, 3H, Ar-*H*), 2.24 (s, 3H, C*H*_3_) ppm; ^13^C-NMR (100 Hz, DMSO-*d*_6_+NaOD): δ = 176.9 (2C), 171.1 (1C), 169.3 (1C), 141.2 (2C), 133.1 (2C), 132.1 (1C), 131.1 (1C), 127.8 (1C), 125.8 (1C), 123.1 (1C), 120.7 (2C), 119.7 (1C), 101.3 (1C), 21.2 (1C) ppm. High-resolution mass spectrum, *m/z*: calculated 373.05912 for C_18_H_14_ClN_2_O_5_ [M + H]^+^; found 373.05899.

#### 3.2.20. *N*-(5-methylbenzoic acid)-4-hydroxy-6-methyl-2-quinolone-3-carboxamide (**25**)

Ethyl 4-hydroxy-6-methyl-2-quinolone-3-carboxylate (**5**) (1 g, 4.044 mmol) and 2-amino-5-methyl benzoic acid (**6s**) (1.83 g, 12.13 mmol) were dissolved in 25 mL THF. A few drops of DMF was added then the mixture was refluxed at 150–160 °C for 48 h. Completion of the reaction was indicated by absence of **5** on TLC and appearance of precipitate at RT. The solid was isolated by filtration, washed with absolute ethanol, and dried under reduced pressure.

A yellow powder (1.2 g) with %yield: 84, R_ƒ_ = 0.6 (CHCl_3_: MeOH-1: 9); m.p: 298–300 °C; ^1^H-NMR (500 Hz, DMSO-*d*_6_): δ = 18.8 (s, 1H, O*H*), 13.1 (s, 1H, COO*H*), 11.76 (s, 1H, N*H*), 10.67 (s, 1H, N*H*), 8.08 (s, 1H, Ar-*H*), 7.07-7.8 (m, 4H, Ar-*H*), 7.07 (s, 1H, Ar-*H*), 2.40 (s, 3H, C*H*_3_), 2.35 (s, 3H, C*H*_3_) ppm; ^13^C-NMR (125 Hz, DMSO-*d*_6_): δ = 176.5 (1C), 173.3 (1C), 172.1 (1C), 170.1 (1C), 146.6 (1C), 137.1 (1C), 130.6 (1C), 130.2 (1C), 130.1 (1C), 129.2 (1C), 129.0 (1C), 125.2 (1C), 124.9 (1C), 123.4 (1C), 122.3 (1C), 121.4 (1C), 102.3 (1C), 21.3 (1C), 20.9 (1C) ppm. High-resolution mass spectrum, *m/z*: calculated 353.11375 for C_19_H_17_N_2_O_5_ [M + H]^+^; found 353.11301.

#### 3.2.21. *N*-(4-mercaptophenyl)-4-hydroxy-6-methyl-2-quinolone-3-carboxamide (**26**)

Ethyl 4-hydroxy-6-methyl-2-quinolone-3-carboxylate (**5**) (1 g, 4.044 mmol) and 4-amino- benzenethiol (**6t**) (1.57 g, 12.52 mmol) were dissolved in 25 mL THF. A few drops of DMF was added then the mixture was refluxed at 150–160 °C for 48 h. Completion of the reaction was indicated by absence of **5** on TLC and appearance of precipitate at RT. The solid was isolated by filtration, washed with absolute ethanol, and dried under reduced pressure.

A yellow powder (1.0 g) with %yield: 84, R_ƒ_ = 0.7 (CHCl_3_: MeOH-9.5: 0.5); m.p: 299–303 °C; ^1^H-NMR (500 Hz, DMSO-*d*_6_+NaOD): δ = 7.66 (s, 1H, Ar-*H*), 7.09 (d, *J* = 8.3 Hz, 1H, Ar-*H*), 6.98 (d, *J* = 8.2 Hz, 4H, Ar-*H*), 6.89 (d, *J* = 8.2 Hz, 1H, Ar-*H*), 2.31 (s, 3H, C*H*_3_) ppm; ^13^C-NMR (125 Hz, DMSO-*d*_6_+NaOD): δ = 177.3 (1C), 176.7 (1C), 171.1 (1C), 168.9 (1C), 147.5 (1C), 144.6 (1C), 133.0 (1C), 132.6 (1C), 131.0 (1C), 125.7 (1C), 125.1 (1C), 122.7 (1C), 120.2 (1C), 119.7 (1C), 119.2 (1C), 100.8 (1C), 21.3 (1C) ppm. High-resolution mass spectrum, *m/z*: calculated 327.08034 for C_17_H_15_N_2_O_3_S [M + H]^+^; found 327.08078.

#### 3.2.22. *N*-(4-(methylthio) phenyl)-4-hydroxy-6-methyl-2-quinolone-3-carboxamide (**27**)

Ethyl 4-hydroxy-6-methyl-2-quinolone-3-carboxylate (**5**) (1 g, 4.044 mmol) and 4-(methylthio) benzenamine (**6u**) (1.68 g, 12.13 mmol) were dissolved in 25 mL THF. A few drops of DMF was added then the mixture was refluxed at 150–160 °C for 48 h. Completion of the reaction was indicated by absence of **5** on TLC and appearance of precipitate at RT. The solid was isolated by filtration, washed with absolute ethanol, and dried under reduced pressure.

A green powder (0.641 g) with %yield: 56, R_ƒ_ = 0.7 (CHCl_3_: MeOH-9.5: 0.5); m.p: 297–299 °C; ^1^H-NMR (500 Hz, DMSO-*d*_6_+NaOD): δ = 14.1 (s, 1H, Ar-O*H*), 7.73 (s, 1H, Ar-*H*), 7.62 (d, *J* = 8.6 Hz, 2H, Ar-*H*), 7.09 (d, *J* = 8.5 Hz, 2H, Ar-*H*), 6.96 (d, *J* = 8.2 Hz, 2H, Ar-*H*), 2.31 (s, 3H, C*H*_3_), 2.12 (s, 3H, C*H*_3_) ppm; ^13^C-NMR (125 Hz, DMSO-*d*_6_+NaOD): δ = 177.5 (1C), 168.5 (1C), 167.1 (1C), 139.2 (1C), 139.0 (1C), 132.0 (1C), 131.9 (1C), 129.5 (1C), 128.2 (2C), 125.7 (1C), 122.1 (1C), 120.6 (1C), 120.5 (1C), 116.20 (1C), 99.3 (1C), 21.2 (1C), 16.6 (1C) ppm. High-resolution mass spectrum, *m/z*: calculated 341.09599 for C_18_H_17_N_2_O_3_S [M + H]^+^; found 341.09519.

### 3.3. Biology

#### 3.3.1. Culture Conditions

Two human colon cancer cell lines; Caco-2 and HCT-116 were maintained in DMEM culture medium (Dulbecco’s modified essential medium, Gibco) (Waltham, MA, USA), supplemented with 5% (*v*/*v*) fetal calf serum (JS Bioscience, Redland, Queensland, Australia), and 1% (*v*/*v*) antibiotic (2 mM L-glutamine, 100 U/mL Penicillin and 0.1 mg/mL Streptomycin; Gibco). Cells were cultured at 37 °C in a humidified 5% CO_2_ incubator. Enzymatic detachment of the confluent cell layers was carried out using Trypsin/ EDTA (Gibco, Waltham, MA, USA), and trypan blue vital staining (0.4% (*w*/*v*); Sigma, USA) was used to assess cell viability with cell number determined using a light microscope.

#### 3.3.2. MTT Assay

All cells were plated at density of 8 × 10^3^ cells per well in 96-well plates and incubated to allow attachment for 24 h. The in vitro evaluation of the antiproliferative activities of the examined series was accomplished using the 3-(4,5-dimethylthiazol-2yl)-2,5-diphenyl tetrazolium bromide (MTT) colorimetric assay, as previously described [41]. In brief, compounds were diluted in culture media to yield the required concentration and applied to test wells for 48 h at 37 °C in a 5% CO_2_ incubator. Three triplicates of each concentration for all tested compounds were evaluated in three independent assays (n = 9). DMEM samples were employed as negative controls, and doxorubicin as a positive control. At the end of the exposure period, 20 µL of 0.5 mg/mL of MTT was added to each well and incubated for 4 h, afterword its reduction to formazan by metabolically active cells was calculated via measuring the absorbance at 570 nm. Cell viability was calculated based on the measured absorbance relative to the absorbance of the cells exposed to the negative control, which represented 100% cell viability.

#### 3.3.3. Statistical Analysis

Data analysis was performed using GraphPad Prism software version 7 (San Diego, CA, USA). The differences between treatment groups were determined by t-test or one-way analysis of variance (ANOVA) followed by Tukey post hoc T-test as appropriate. Data were expressed as mean ± SD and *p* < 0.05 was considered a statistically significant difference. A non-linear regression analysis was used to calculate IC_50_ values.

#### 3.3.4. Quantitative Real-Time PCR

##### RNA Extraction

Total RNA was extracted from the cultured cells using Quick-RNA MiniPrep according to the manufacturer’s instructions as follows: harvested cells were re-suspended in RNA lysis buffer and centrifuged, the supernatant then was transferred into a Spin-Away Filter in a collection tube and centrifuged, and then 95% ethanol was added and mixed well. The mixture was then transferred to a Zymo-Spin IIICG Column in a collection tube and centrifuged, followed by adding RNA Prep Buffer to the column, followed by two washing steps by adding RNA wash buffer to the column and then centrifuged. Finally, the RNA was eluted by adding DNase/RNase-free water to the column and it was centrifuged. Samples were quantified using a spectrophotometer via absorbance at 260/280 nm.

##### Complementary DNA (cDNA) Synthesis

Complementary cDNA synthesis was performed using ProtoScript First Strand cDNA Synthesis Kit following the manufacturer’s instructions. In brief: 1000 ng from total RNA was added to a total of 20 µL reaction volume that included 60 µM random primer mix, reaction mix, 10× enzyme mix, and nuclease-free water. The tubes were then incubated at 25 °C for 5 min followed by incubation at 42 °C for 1 h. The cDNA was then then stored at −20 °C until further analysis.

##### Real-Time PCR

The sequences of the primers that were used in real-time PCR assay are shown in Table 4. The reaction mixtures consisted of 200 ng cDNA template, 10 µM of each primer, 10 µL of 2× SYBER Premix Ex Taq II (Takara BIO INC, Shiga, Japan), and the total reaction volume was 20 µL. The reaction was carried out using a BIO RAD iQ5 Multicolor real-time PCR Detection System thermal cycler under the following reaction conditions: 1 cycle of 2 min at 95.0 °C, followed by 45 cycles of 10 s at 95.0 °C, 25 s at 57.0 °C, 25 s at 60.0 °C, and a final cycle of 30 s at 55.0 °C. To confirm that only one PCR product was amplified, dissociation curve analysis of amplification products was performed at the end of the last amplification cycle.

### 3.4. Computational Methods

#### 3.4.1. Preparation of PI3Kα Structure

The X-ray structures of WT PI3Kα (PDB ID: 2RD0) [4] and (PDB ID: 4L23) [33] as well as MUT (H1047R) PI3Kα (PDB ID: 3HHM) [29] were retrieved from the RCSB Protein Data Bank. The coordinates of X6K in 4L23 [33] were transferred to 2RD0 and assigned as a ligand to derive the grid file. The homology modeled structures of 2RD0 and 3HHM were adopted for this study [14]. Energy minimization was employed to reduce steric clash.

Further treatment of the minimized structures was performed using Protein Preparation module in Schrödinger enterprise [32] to optimize H-bond interactions between backbones.

#### 3.4.2. Preparation of Ligand Structures

The synthesized compounds (ligands) were built using wortmannin’s coordinates in 3HHM. The ligands were modeled using MAESTRO [32] Build wizard and energetically minimized by the MacroModel module using OPLS2005 force field.

#### 3.4.3. Induced-Fit Docking (IFD)

The co-crystallized ligand (wortmannin) was assigned as a centroid in the kinase binding site of 2RD0 [4] and 3HMM [29]. The Vander Waals scaling factors for receptor and ligand were adjusted to 0.5 to furnish flexibility for the best docked ligand conformation. Other parameters were set as default. The ligand pose with the highest XP Glide score was identified.

#### 3.4.4. Molecular Descriptors

All molecular structures, sketched in ChemDraw [42] and saved in SDF file format, were standardized according to the methods described by Hajjo et al. [43]. Next, two groups of molecular descriptors, comprising ‘Drug-like Indices’ and ‘Molecular Properties’ calculated using alvaDesc software from Kode Cheminformatics [40], were generated for compounds **5**, **7**–**27**.

#### 3.4.5. Principal Component Analysis (PCA)

A principal component analysis was performed on structures **5** and **7**–**27** using drug-like indices and molecular properties. All calculations and PCA analysis were performed using alvaDesc software from Kode Cheminformatics [40].

## 4. Conclusions

Phosphatidylinositol 3-kinase (PI3Kα) has been underlined as a potential target for anticancer drug design. We identified a series of *N*-phenyl-4-hydroxy-6-methyl-2-quinolone-3-carboxamide as possible PI3Ká inhibitors. Biological evaluation showed that the series exhibited high inhibitory activity against Caco-2 and HCT-116 cell lines. Results revealed that compound **16** has a substantial effect on AKT, BAD, and PI3K gene expression. Docking studies against PI3Kαs displayed that the scaffold orientates in PI3Kαs kinase domains and form H-bonds with key binding residues. We are looking to optimize the core structure of this series to induce its anticancer activity and selectivity against a panel of kinases.

## Figures and Tables

**Table 1:**
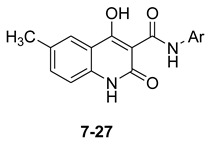
Chemical structures of synthesized *N*-substituted-4-hydroxy-6-methyl-2-quinolone-3- carboxamides (**7**–**27**).

Compound	Ar	Compound	Ar
**7**	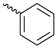	**18**	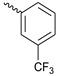
**8**	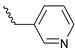	**19**	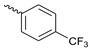
**9**	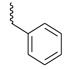	**20**	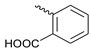
**10**	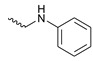	**21**	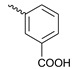
**11**	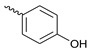	**22**	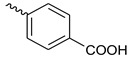
**12**	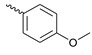	**23**	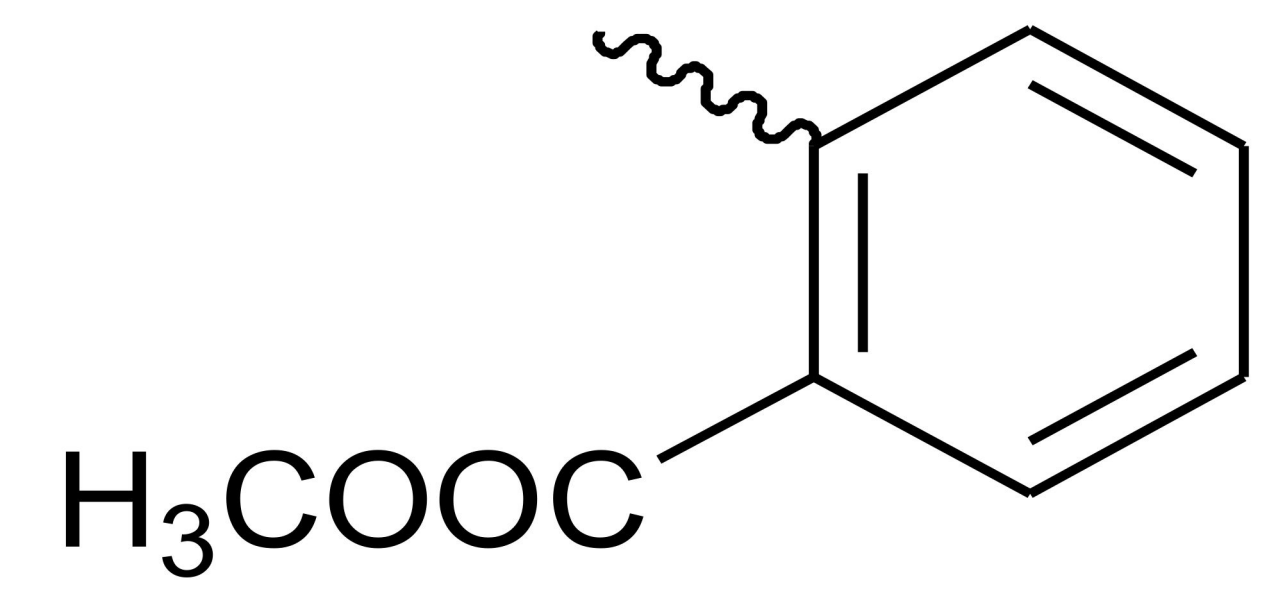
**13**	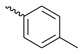	**24**	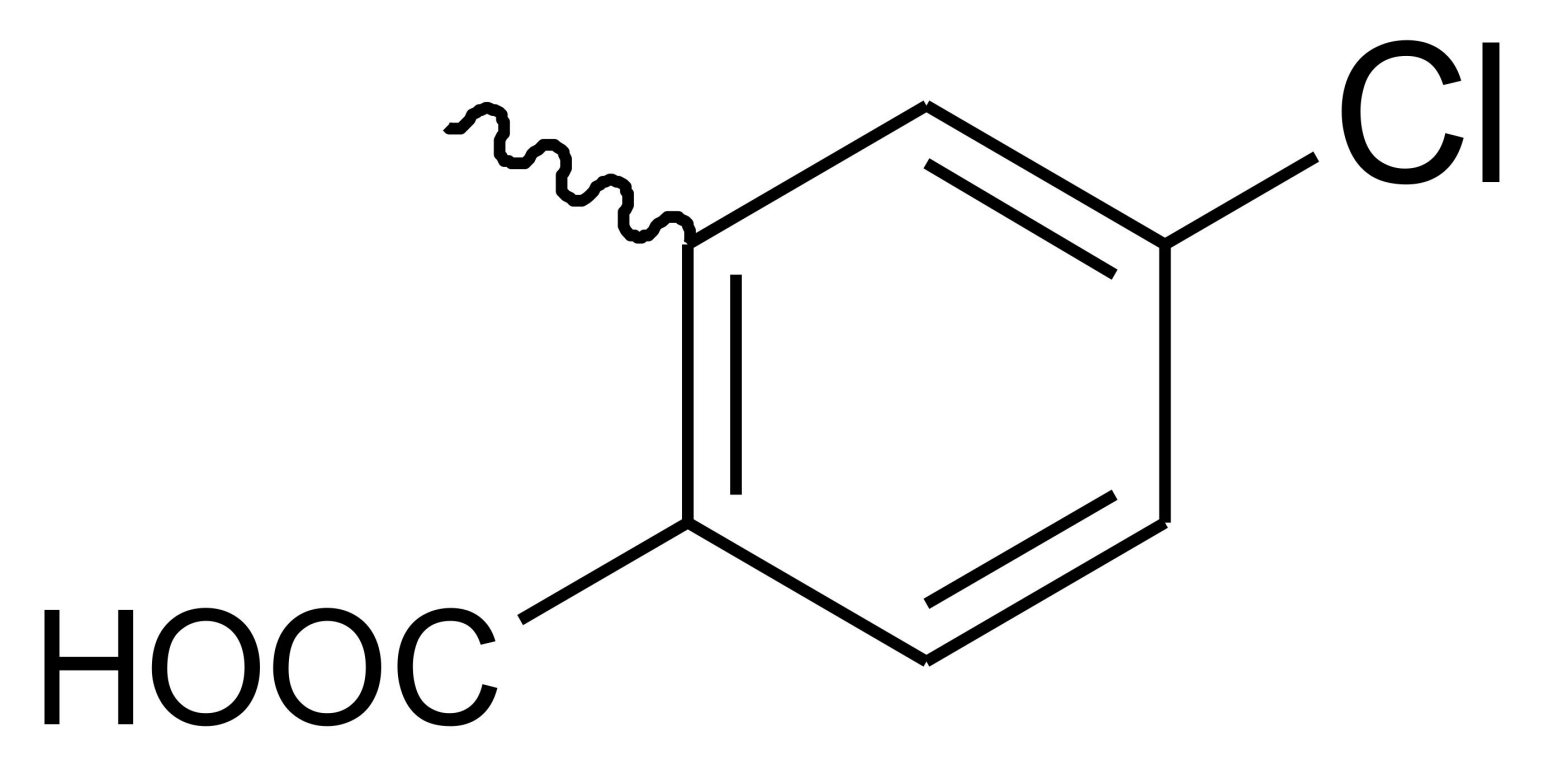
**14**	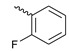	**25**	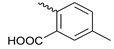
**15**	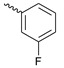	**26**	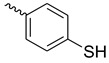
**16**	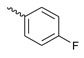	**27**	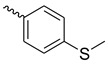
**17**	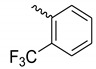		

**Table 2:** The inhibitory activity (IC_50_ µM) of **5**, **7**–**27**. SD does not exceed 5%.

Compound’s Number	IC_50_ (µM) ± SD		Compound’s Number	IC_50_ (µM) ± SD	
	Caco-2	HCT-116		Caco-2	HCT-116
LY294002	7.4	6.5	**17**	2321	615
5	338.5	346.8	**18**	486.6	422.5
7	264	212.2	**19**	402.3	304.2
8	98	337	**20**	1559	789.4
9	356	383.7	**21**	664.5	16.04
10	275	390.4	**22**	458.2	184.6
11	204	92.61	**23**	261.4	46.38
12	260	207.4	**24**	298	241.1
13	687	178.6	**25**	302	140.0
14	354	441.2	**26**	282.5	405.7
15	212	282.2	**27**	289.2	462.7
16	13	240.2			

**Table 3:** The IFD scores (Kcal/mol) and H-bond interactions between 2RD0 and 3HHM.

	2RD0		3HHM	
Compound	Docking Score	Binding Residues	Docking Score	Binding Residues
**5**	−7.58	K802, D933	−7.77	V851
**7**	−8.04	V851	−8.91	S774, D933, N920
**8**	−7.84	S774, D933	−8.73	S774, D933, S919
**9**	−8.98	E849, V851	−8.71	S774, D933
**10**	−9.39	W780, E849, V851, S854	−8.72	S774, D933, S919, N920
**11**	−9.40	V851	−9.68	S774, D933, S919, F1059
**12**	−8.69	V851, Q859	−9.32	Y836, V851
**13**	−8.59	V851	−7.92	S774, D933
**14**	−8.37	Y836, D933	−8.09	S774, S919, D933
**15**	−8.79	V851	−8.99	S774, S919, D933
**16**	−9.20	W780, V851	−8.41	Y836
**17**	−8.44	S854, Q859	−8.39	N920, D933
**18**	−8.65	W780, V851	−8.33	S774, D933, N920
**19**	−8.42	K802, D933	−8.95	S773, D933
**20**	−8.89	S774, K802, D933	−9.98	S774, A775, K776, D933, N920
**21**	−9.15	S774, K776, D805, D933	−9.08	S774, D915, H917, N920, D933
**22**	−10.32	K802, D810, V851	−9.58	S774, E849, D933
**23**	−8.11	S774, K802, D933	−8.47	S774, N920
**24**	−8.39	W780, S854, Q859	−9.46	S774, H917, N920
**25**	−9.83	V851, S854, D933	−10.04	S774, K776, N920, D933
**26**	−8.59	D933	−8.52	S773, N920
**27**	−8.81	V851	−10.0	D810, Y836, D933

**Table 4:** Sequences of primers that were used to detect the expression of β-actin, BAD, PI3K, and AKT.

Target	Forward Primers (5′→3′)	Reverse Primers (5′→3′)
β-actin	ACGGGGTCACCCACACTGTGC	CTAGAAGCATTTGCGGTGGACGATG
BAD	CCTCAGGCCTATGCAAAAAG	AAACCCAAAACTTCCGATGG
PI3K	ACCCAGCAACAGAAAAATGG	GCGCTGTGAATTTAGCCTTC
AKT	AACCTGTGCTCCATGACCTC	CCCTTCTACAACCAGGACCA

## Supplementary Materials

The following are available online, Table S1: Drug-like and alvaDesc molecular properties.
